# Functional Characterizations of *RIG*-*I* to GCRV and Viral/Bacterial PAMPs in Grass Carp *Ctenopharyngodon idella*

**DOI:** 10.1371/journal.pone.0042182

**Published:** 2012-07-31

**Authors:** Lijun Chen, Jianguo Su, Chunrong Yang, Limin Peng, Quanyuan Wan, Lan Wang

**Affiliations:** College of Animal Science and Technology, Northwest A&F University, Shaanxi Key Laboratory of Molecular Biology for Agriculture, Yangling, People’s Republic of China; Auburn University, United States of America

## Abstract

**Background:**

*RIG-I* (*retinoic acid inducible gene-I*) is one of the key cytosolic pattern recognition receptors (PRRs) for detecting nucleotide pathogen associated molecular patterns (PAMPs) and mediating the induction of type I interferon and inflammatory cytokines in innate immune response. Though the mechanism is well characterized in mammals, the study of the accurate function of *RIG-I* in teleosts is still in its infancy.

**Methodology/Principal Findings:**

To clarify the functional characterizations of *RIG-I* in grass carp *Ctenopharyngodon idella* (*CiRIG-I*), six representative overexpression plasmids were constructed and transfected into *C. idella* kidney (CIK) cell lines to obtain stably expressing recombinant proteins, respectively. A virus titer test and 96-well plate staining assay showed that all constructs exhibited the antiviral activity somewhat. The quantitative real-time RT-PCR (qRT-PCR) demonstrated that mRNA expressions of *CiIPS-1*, *CiIFN-I* and *CiMx2* were regulated by not only virus (GCRV) or viral PAMP (poly(IC)) challenge but also bacterial PAMPs (LPS and PGN) stimulation in the steadily transfected cells. The results showed that the full-length *CiRIG-I* played a key role in RLR pathway. The repressor domain (RD) exerted an inhibitory function of the signaling channel under all utilized challenges. Caspase activation and recruitment domains (CARDs) showed a positive role in GCRV and poly(I:C) challenge. Helicase motifs were crucial for the signaling pathway upon LPS and PGN stimulation. Interestingly, ΔCARDs (CARDs deleted) showed postive modulation in *RIG-I* signal transduction.

**Conclusions/Significance:**

The results provided some novel insights into *RIG-I* sensing with a strikingly broad regulation in teleosts, responding not only to the dsRNA virus or synthetic dsRNA but also bacterial PAMPs.

## Introduction

The innate immune system serves as the first line of protection against invading microbial pathogens through a limited number of germ line-encoded pattern recognition receptors (PRRs) [1]. The PRRs recognize different but overlapping pathogen-associated molecular patterns (PAMPs), and trigger innate immune responses and subsequent adaptive immunity [2]. Currently, four major classes of PRRs have been identified and classified into transmembrane proteins such as C-type lectin receptors (CLRs) and Toll-like receptors (TLRs), and cytoplasmic proteins such as NOD-like receptors (NLRs) and RIG-I-like receptors (RLRs) [3]. The innate immune system recognizes viral nucleic acids and microbial pathogens mainly by the latter three families [4]. TLRs are expressed in cell surfaces or in endosomes, and they recognize virus, bacteria, fungi and protozoa [5]. NLRs mainly detect pathogenic bacteria [6]. In contrast, RLRs primarily sense virus-derived RNA molecules in the cytoplasm [7], [8]. Upon activation, TLRs and RLRs trigger *type I interferon* (*IFN-I*), leading to an enhanced antiviral state of host cells [9].

RLRs are composed of three members: *retinoic acid-inducible gene I* (*RIG-I*, also known as *DDX58*), *melanoma differentiation-associated gene 5* (*MDA5*, also known as *IFIH1* or *Helicard*), and *laboratory of genetics and physiology 2* (*LGP2*, also known as *DHX58*). RLRs evolve from a common ancestor encoding different core functional domains between mammals and teleosts [10], [11]. Recently, the RLR family has become a major focus on the research of innate immunity [8], [12]. *RIG-I* is a particular sensor in nucleotide recognition, and the regulatory functions of *RIG-I* are broad, not only in detection of RNA and DNA viruses but in recognition of bacterial component (such as lipopolysaccharide (LPS)) as well [13], [14].

As is well known, *RIG-I* consists of three distinct domains: (1) N-terminal two tandem caspase activation and recruitment domains (CARDs), (2) central DExD/H box RNA helicase domain, and (3) C-terminal regulatory/repressor domain (RD) [15]. The CARDs of *RIG-I* mediate the interaction with the CARD of *interferon-β promoter stimulator 1* (*IPS-1*; also known as *MAVS*, *CARDIF*, or *VISA*) [16]. *IPS-1* functions as an adaptor molecule, linking the sensors of *RIG-I* to the kinases *TBK1* (*TANK-binding kinase 1*) and *IKK-ε* (*inhibitor of nuclear factor Iκ kinase-ε*), which phosphorylate *interferon regulatory factors-3/7* (*IRF-3/7*). Upon phosphorylation, *IRF-3/7* dimerizes, then translocates into the nucleus, and subsequently induces *IFN-I* and *ISGs* (*IFN-stimulated genes*) expressions [17]. *IFN-I* is also induced by PAMPs, such as LPS, peptidoglycan (PGN) and CpGDNA, and it plays a key role in limiting the spread of pathogens [18]. *ISGs* include *double-stranded RNA-activated protein kinase* (*PKR*), 2′,5′-*oligoadenylate synthetase* (*OAS*), *inducible nitric oxide synthase* (*iNOS*), *RNA-specific adenosine deaminase* (*ADAR*) and *myxovirus-resistant protein* (*Mx*) amongst others [19], [20].

In mammals, functional domains and signaling pathways of *RIG-I* have been extensively studied [21]–[23]. Recently, *RIG-I* has been identified in many fish species and expression characterizations are also shown in zebrafish, fathead minnow, Atlantic salmon, grass carp, common carp, crucian carp and channel catfish [24]–[29]. However, little is known about the functions of *RIG-I* domains in teleosts [24].

LPS is an outer-membrane component of gram-negative bacteria that can be recognized by *TLR4* [30], and PGN is a major element of gram-positive bacterial cell walls that can be recognized by *TLR2*, *NOD1* and *NOD2* [31]. Since eukaryotic organisms do not contain LPS and PGN in their cellular structures, LPS and PGN are ideal target molecules for detecting bacterial invasion. It has been evidenced that *RIG-I* is induced in endothelial cells and macrophages post LPS stimulation [14], [32].

Grass carp (*Ctenopharyngodon idella*) is employed as a model for antiviral immune studies because it is a crucial aquaculture species in China and is susceptible to grass carp reovirus (GCRV), a double-stranded RNA (dsRNA) virus [33]. Better understanding of the immune defense mechanisms may be conducive to the development of management strategies for disease control and comprehensive study on innate immune system evolution in teleosts [10], [34].

To research functional characterizations of *RIG-I* in grass carp (*CiRIG-I*), six overexpression vectors were constructed, including full-length *CiRIG-I* and a series of domain containers, and they were transfected into *C. idella* kidney (CIK) cell line to obtain steadily expressing recombinant proteins. The mRNA expressions of downstream genes (*CiIPS-1*, *CiIFN-I* and *CiMx2*) of *CiRIG-I* were examined post GCRV and poly(I:C) (polyinosine-polycytidylic acid, a synthetic analog of dsRNA) challenges, as well as LPS and PGN stimulation.

## Results

### Antiviral Activity of CiRIG-I and its Domains

**Figure 1:**
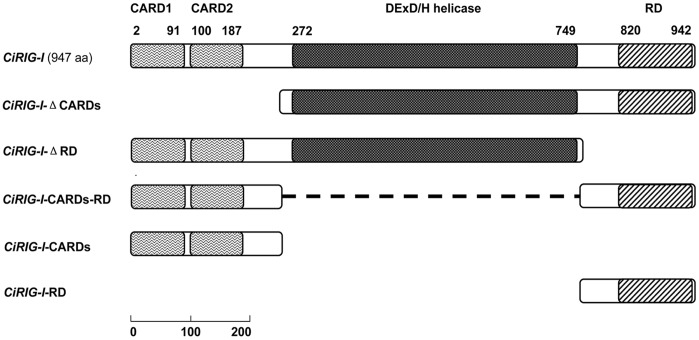
Schematic representation of the full length and domains of *CiRIG-I*. The full-length *CiRIG-I* consists of two CARDs, a DExD/H helicase and a RD. To investigate the functions of pivotal domains of *CiRIG-I*, a series of plasmids were constructed, including the full-length *CiRIG-I* (1–947, 947 aa); *CiRIG-I-*ΔCARDs (249–947, 697 aa); *CiRIG-I-*ΔRD (1–758, 758 aa); *CiRIG-I-*CARDs-RD (1–253 and 756–947, 445 aa); *CiRIG-I-*CARDs (1–254, 254 aa); *CiRIG-I-*RD (755–947, 193 aa). For detailed representation: aa (amino acids), CARDs (2–91, 100–187 aa), helicase domain (272–749 aa), RD (820–942 aa). The scale was shown at the bottom left corner.

**Figure 2:**
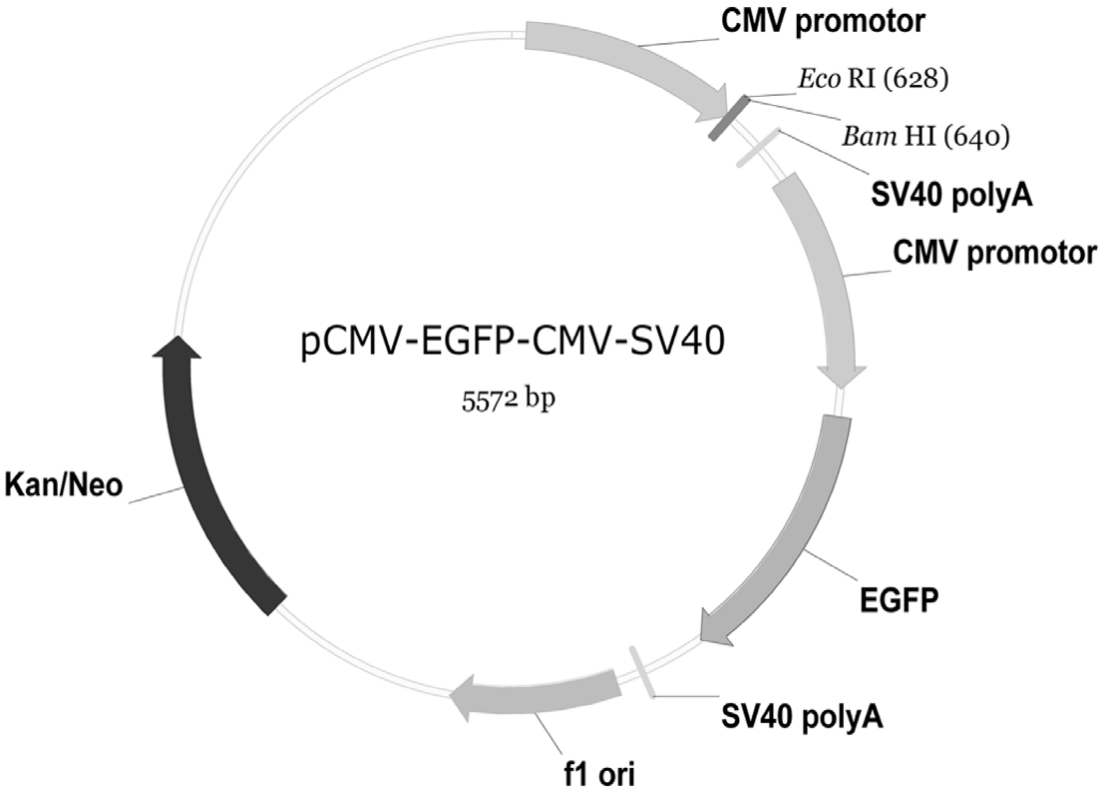
Illustration of pCMV-EGFP-CMV-SV40 plasmid. It contains additional CMV promoter and SV40 polyA transcription termination sequence. It holds kanamycin (Kan) or neomycin (Neo) selection sequence, restriction enzyme sites (*Eco*RI and *Bam*HI), and the skeleton component of original pCMV-EGFP plasmid.

**Figure 3:**
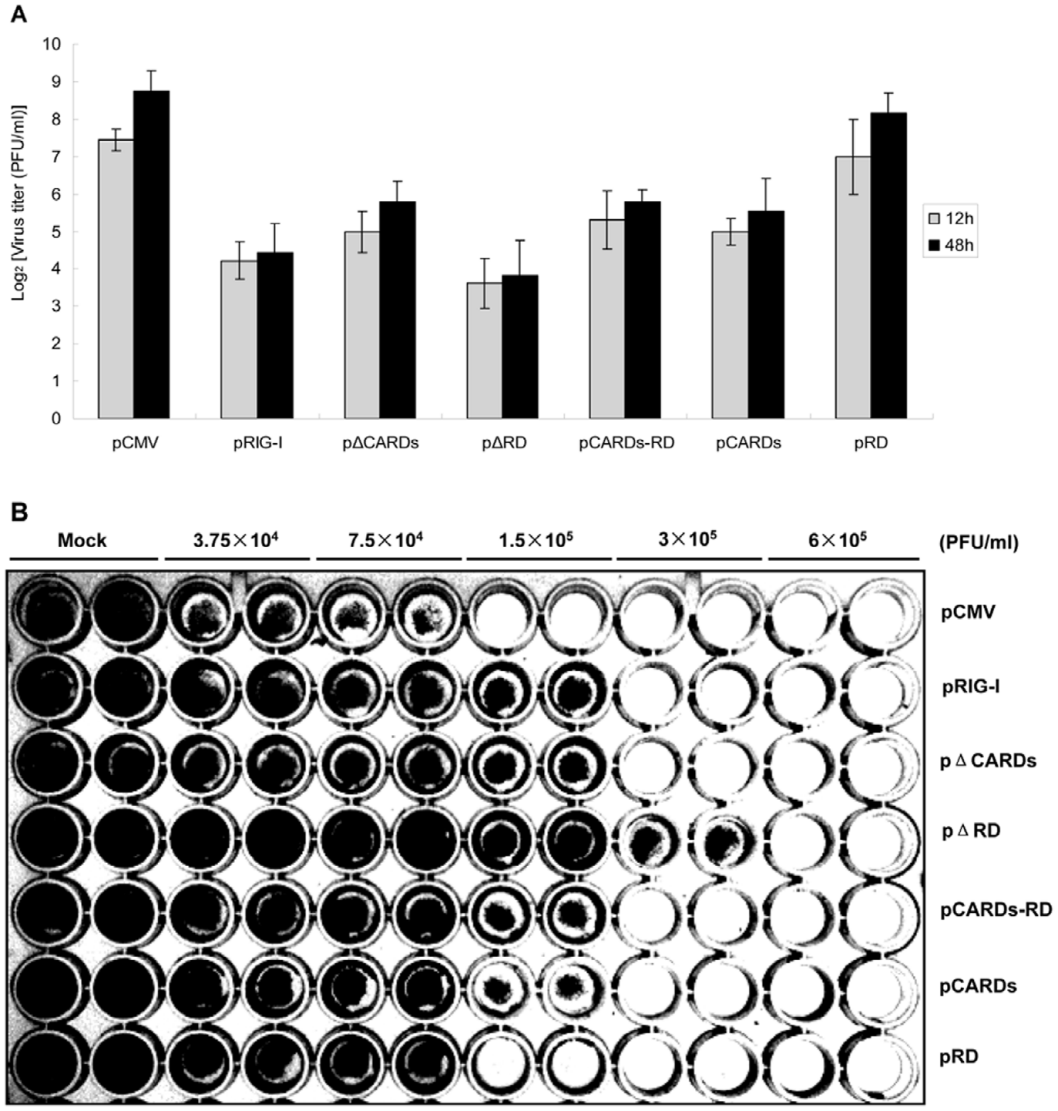
Virus titer test and antiviral activity assay of *CiRIG-I*. (A) Virus titer was checked in transfected cells, including pCMV (control) and six representative RIG-I variants by plaque assay. Fresh CIK cells were seeded in 96-well plates, and they were inoculated with 2-fold serial dilution of transfected cell supernatants (harvested at 12 and 48 h) in duplicate. At 72 h post infection, the cells were observed and the viral titer was calculated. The results represented two individual experiments, and error bars indicated the standard deviation (SD). (B) In antiviral activity assay, the steadily transfected cells were seeded into a 96-well plate, then infected with 2-fold-diluted GCRV in duplicate. At 60 h post infection, cells were fixed with 10% paraformaldehyde for 10 min at room temperature, and then stained with 0.05% (wt/vol) crystal violet for 30 min. Washed with water and drained, and the plates were photographed under a light box.

To clarify the functional characterizations of *CiRIG-I*, six representative overexpression plasmids were constructed (Fig. 1) for stably expressing recombinant protein in CIK cells, respectively. The pCMV (Fig. 2) was employed as a control. Measurement of the viral titer showed that overexpression of *CiRIG-I* and its variants decreased the viral titer more or less compared to that in control cells (Fig. 3A). Overexpression of *CiRIG-I* decreased the viral titers of 9-fold and 20-fold at 12 h and 48 h, respectively. Similarly, the viral titers in cells transfected with pΔRD reduced 14-fold at 12 h and 31-fold at 48 h. The viral titers in cells transfectected with pΔCARDs, pCARDs-RD and pCARDs also declined 4–5 folds at 12 h and 7–9 folds and at 48 h, respectively. As for the viral titers in cells transfected with pRD, they showed slight descent. The consistent results were obtained by antiviral activity assay (Fig. 3B). The cells transfected with pΔRD and pRIG-I exhibited the powerful antiviral activity. The cells transfected with pΔCARDs, pCARDs-RD and pCARDs displayed moderate roles of resistance to GCRV. The cells transfected with pRD owned little inhibitory effect (Fig. 3B).

### CARDs and Helicase of CiRIG-I Participate in the Signaling Cascade after GCRV Infection, Whereas RD Inhibits the Activation

**Figure 4:**
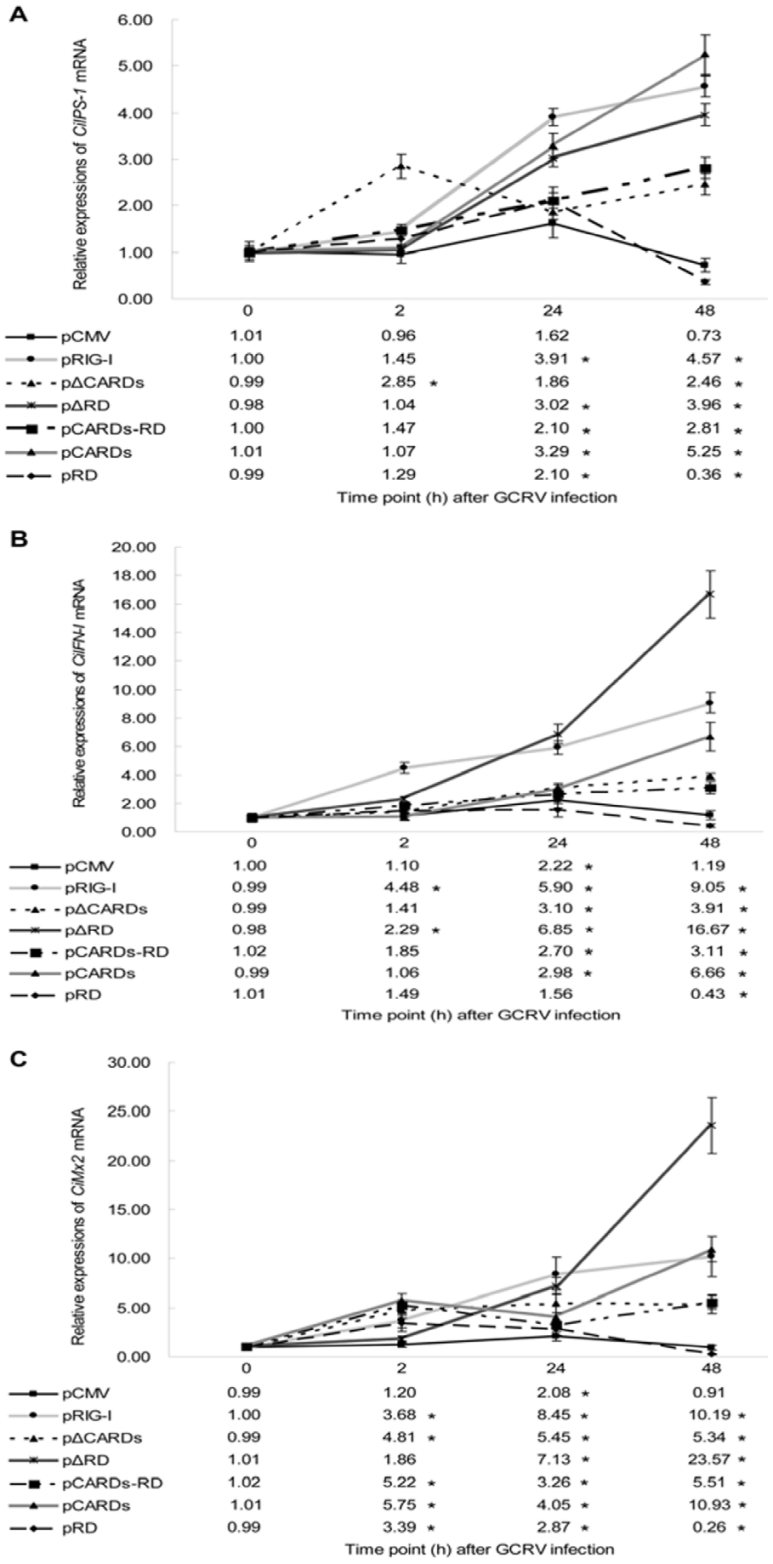
The mRNA expressions of several downstream genes of *CiRIG-I* post GCRV infection in seven stable transgenic cells. These genes included *CiIPS-1* (A), *CiIFN-I* (B) and *CiMx2* (C). Seven constructs include pCMV (control), pRIG-I, pΔCARDs, pΔRD, pCARDs-RD, pCARDs and pRD. The mRNA expressions were measured at 0, 2, 24 and 48 h post GCRV challenge. The *EF1α* gene was used as an internal control to normalize the cDNA template. Error bars indicated SD. Asterisk (*) was marked significant difference (P<0.05) between experimental group and control group. Detailed values were listed at the bottom of the figure.

The mRNA expression patterns of *CiIPS-1* (Fig. 4A), *CiIFN-I* (Fig. 4B) and *CiMx2* (Fig. 4C) were revealed by quantitative real-time RT-PCR (qRT-PCR) in steadily transfected cells after GCRV infection. According to the expression profiles of *CiIPS-1* (Fig. 4A), the relative values in cells transfected with pRIG-I were up-regulated, reached 3.91 folds at 24 h and 4.57 folds at 48 h. The relative quantities in cells transfected with pΔCARDs were slightly increased at 2 h (2.85 folds) and recovered the control level at 24 h, then were slightly enhanced at 48 h (2.46 folds). The relative values in cells transfected with pΔRD were enhanced at 24 h (3.02 folds) and 48 h (3.96 folds). The relative folds in cells transfected with pCARDs-RD were slightly increased at 24 h (2.10 folds) and 48 h (2.81 folds). The relative quantities in cells transfected with pCARDs were enhanced at 24 h (3.29 folds) and 48 h (5.25 folds).The relative values in cells transfected with pRD were slightly increased at 24 h (2.10 folds), then decreased at 48 h (0.36 fold).

As for the expression profiles of *CiIFN-I* (Fig. 4B), the relative values in cells transfected with pRIG-I were enhanced, reached 4.48 folds at 2 h, 5.90 folds at 24 h, and 9.05 folds at 48 h compared to the control. The relative quantities in cells transfected with pΔCARDs were enhanced at 24 h (3.10 folds) and 48 h (3.91 folds). The relative values in cells transfected with pΔRD reached 2.29 folds at 2 h, 6.85 folds at 24 h and 16.67 folds at 48 h. The relative quantities in cells transfected with pCARDs-RD were slightly increased at 24 h (2.70 folds) and 48 h (3.11 folds). Just like the expression model in cells transfected with pΔCARDs, the folds in cells transfected with pCARDs were significantly enhanced at 24 h (2.98 folds) and 48 h (6.66 folds). The relative values in cells transfected with pRD were no significant differences at 2 h and 24 h, then significantly decreased at 48 h (0.43 fold).

The temporary expression patterns of *CiMx2* (Fig. 4C) after GCRV infection were researched. The relative values in cells transfected with pRIG-I reached 3.68 folds at 2 h, 8.45 folds at 24 h and 10.19 folds at 48 h compared to the control. The relative quantities in cells transfected with pΔCARDs were enhanced at 2 h (4.81 folds), maintaining the high folds of 5.45 at 24 h and 5.34 at 48 h. The relative expressions in cells transfected with pΔRD were significantly enhanced at 24 h (7.13 folds) and 48 h (23.57 folds). Similar to transfected pΔCARDs cells, the relative values in cells transfected with pCARDs-RD were significantly up-regulated at 2 h (5.22 folds), maintaining high levels at 24 h (3.26 folds) and 48 h (5.51 folds). Just like transfected pRIG-I cells, the folds in cells transfected with pCARDs were significantly increased at 2 h (5.75 folds), 24 h (4.05 folds) and 48 h (10.93 folds). The relative expressions in cells transfected with pRD were slightly increased at 2 h (3.39 folds) and 24 h (2.87 folds), then sharply decreased at 48 h (0.26 fold).

These results indicated that CARDs and helicase were crucial for antiviral activity, and RD inhibited the activation of *RIG-I* signaling cascade. Unexpectedly, the ΔCARDs significantly enhanced the signaling channel to inhibit GCRV replication.

### Relative Quantities of GCRV Decreased in pΔCARDs Transfected Cells

**Figure 5:**
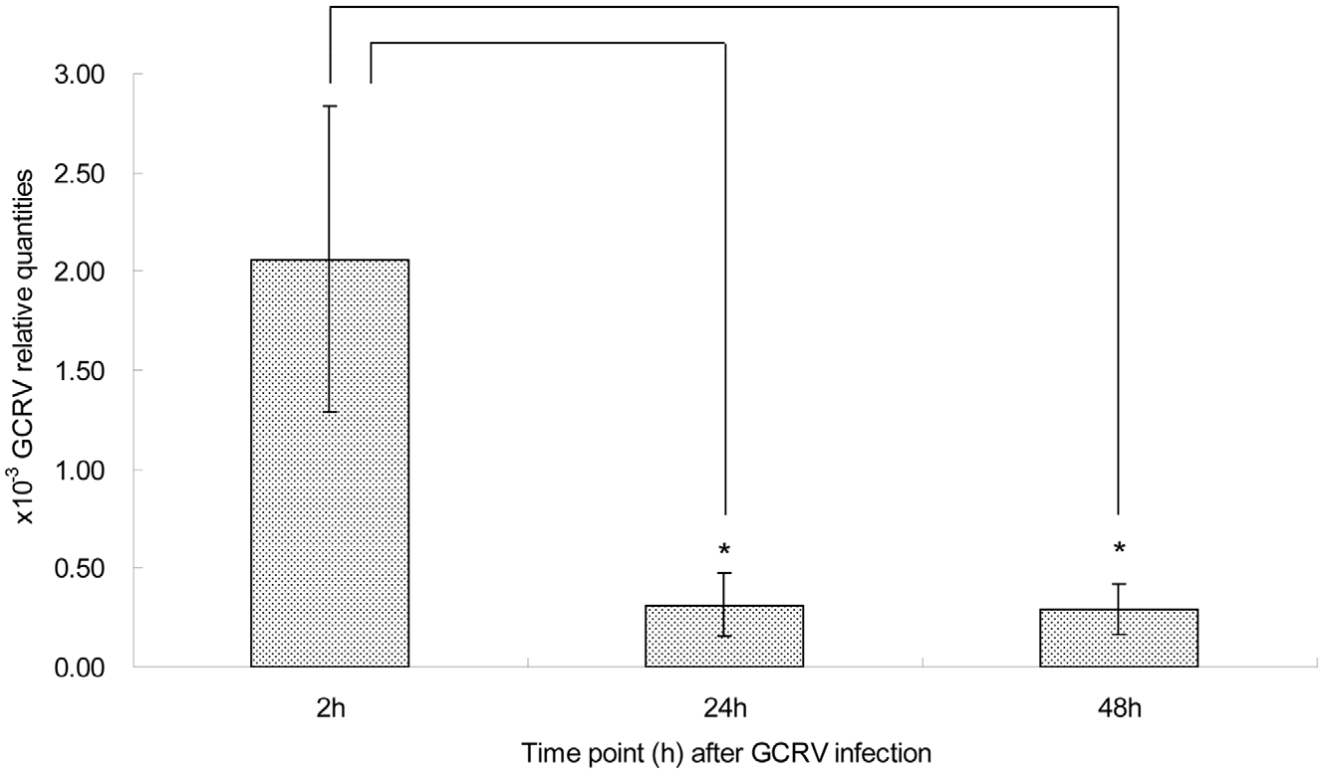
The relative virus quantities in pΔCARDs transfected cells post GCRV infection. They were measured at 2, 24 and 48 h post stimulation. The GCRV quantities in pΔCARDs tranfected cells were relative to those in pCMV transfected cells. Error bars indicated SD. Asterisks (*) indicated significant differences between the pCMV and pΔCARDs transfected cells at indicated time points.

Further experiment was conducted to investigate the antiviral effect in the pΔCARDs and pCMV (control) transfected cells. The GCRV quantities (Fig. 5) were examined at 2, 24 and 48 h by qRT-PCR. And the relative values in cells transfected with pΔCARDs were 2.06×10^−3^ at 2 h, then declined to 0.32×10^−3^ at 24 h and 0.29×10^−3^ at 48 h. The data demonstrated that ΔCARDs of *CiRIG-I* were significantly resistant to GCRV replication.

### CARDs of CiRIG-I Mediate Signaling Function Post Poly(I:C) Stimulation, and RD Prevents this Activation

**Figure 6:**
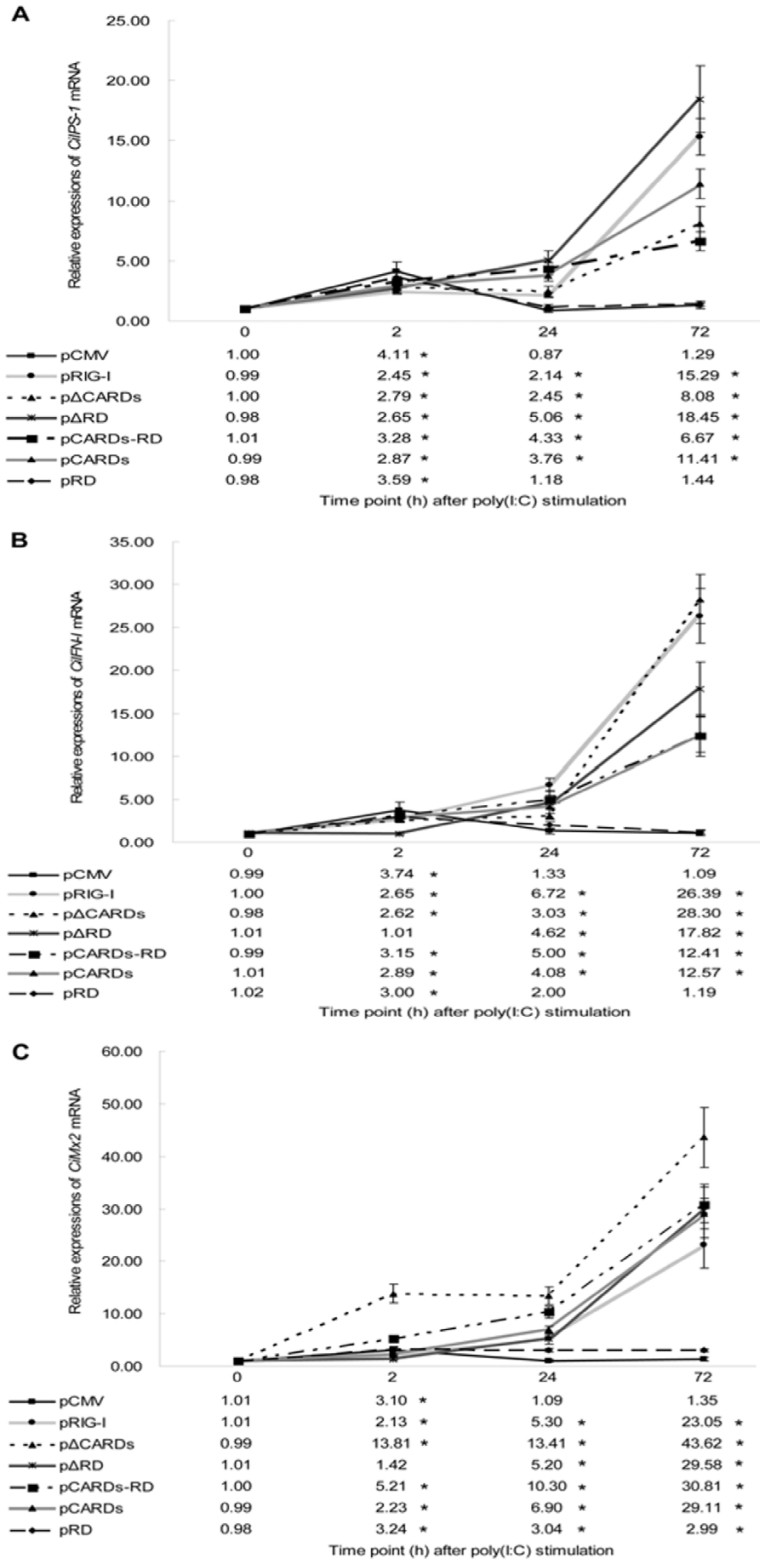
The mRNA expression patterns of several downstream genes of *CiRIG-I* post poly(I:C) stimulation in seven stable transgenic cells. These genes included *CiIPS-1* (A), *CiIFN-I* (B) and *CiMx2* (C). The mRNA expressions were measured at 0, 2, 24 and 72 h post stimulation and the concentration of poly(I:C) was 5 µg/ml. Other captions were the same as Fig. 4.

To further investigate antiviral effects of *CiRIG-I* and its variants, the mRNA expressions of *CiIPS-1* (Fig. 6A), *CiIFN-I* (Fig. 6B) and *CiMx2* (Fig. 6C) were examined at 0, 2, 24 and 72 h post poly(I:C) stimulation. According to the expression profiles of *CiIPS-1* (Fig. 6A), the relative values in cells transfected with pRIG-I were slightly increased at 2 h (2.45 folds) and 24 h (2.14 folds), then rapidly enhanced at 72 h (15.29 folds) compared to the control. Similarly, the relative expressions in cells transfected with pΔCARDs were slightly increased at 2 h (2.79 folds) and 24 h (2.45 folds), then reached the peak at 72 h (8.08 folds). The relative quantities in cells transfected with pΔRD reached 2.65 folds at 2 h, 5.06 folds at 24 h and 18.45 folds at 72 h. The temporal expressions in cells transfected with pCARDs-RD were increased moderately, and the relative values were 3.28 folds at 2 h, 4.33 folds at 24 h and 6.67 folds at 72 h. Just like the transfected pΔCARDs cells, the relative expressions in cells transfected with pCARDs were slightly increased at 2 h (2.87 folds) and 24 h (3.76 folds), and rapidly enhanced at 72 h (11.41 folds). The relative quantities in cells transfected with pRD were increased at 2 h (3.59 folds), then recovered the control level at 24 h and 72 h.

As for the expression profiles of *CiIFN-I* (Fig. 6B), the relative values in cells transfected with pRIG-I reached 2.65 folds at 2 h, 6.72 folds at 24 h and 26.39 folds at 72 h compared to the control. The relative quantities in cells transfected with pΔCARDs were slightly increased at 2 h (2.62 folds) and 24 h (3.03 folds), then sharply enhanced at 72 h (28.30 folds). The relative expressions in cells transfected with pΔRD were no significant differences at 2 h, and rapidly increased at 24 h (4.62 folds) and 72 h (17.82 folds). Similar to the transfected pRIG-I cells, the relative quantities in cells transfected with pCARDs-RD were significantly enhanced at 2 h (3.15 folds), 24 h (5.00 folds) and 72 h (12.41 folds). Like the pattern in transfected pCARDs-RD cells, the fold changes in cells transfected with pCARDs were increased at 2 h (2.89 folds), 24 h (4.08 folds) and at 72 h (12.57 folds), respectively. The relative quantities in cells transfected with pRD were slightly increased at 2 h (3.00 folds) and 24 h (2.00 folds), then retrieved the control level at 72 h.

Next, the temporary expression patterns of *CiMx2* (Fig. 6C) was examined. The relative values in cells transfected with pRIG-I reached 2.13 folds at 2 h, 5.30 folds at 24 h and 23.05 folds at 72 h compared to the control. The relative quantities in cells transfected with pΔCARDs were significantly enhanced at 2 h (13.81 folds), maintained high levels at 24 h (13.41 folds) and 72 h (43.62 folds). The fold changes in cells transfected with pΔRD were significantly enhanced at 24 h (5.20 folds) and reached the peak at 72 h (29.58 folds). Similar to the transfected pΔCARDs cells, the relative values in cells transfected with pCARDs-RD were significantly up-regulated at 2 h (5.21 folds), maintained high levels of 10.30 folds at 24 h and 30.81 folds at 72 h. Just like the transfected pRIG-I cells, the relative quantities in cells transfected with pCARDs were enhanced at 2 h (2.23 folds), 24 h (6.90 folds) and 72 h (29.11 folds). The relative values in cells transfected with pRD were increased at 2 h (3.24 folds), then maintained approximate 3 folds at 24 h and 72 h.

These results showed the CARDs were pivotal for signaling cascade upon poly(I:C) stimulation, and the RD played a negative role. As for helicase, it was not significant for *RIG-I* signaling pathway.

### Helicase Domain of CiRIG-I Mediates Signaling Function upon LPS Stimulation and CARDs Play an Assistant Role

**Figure 7:**
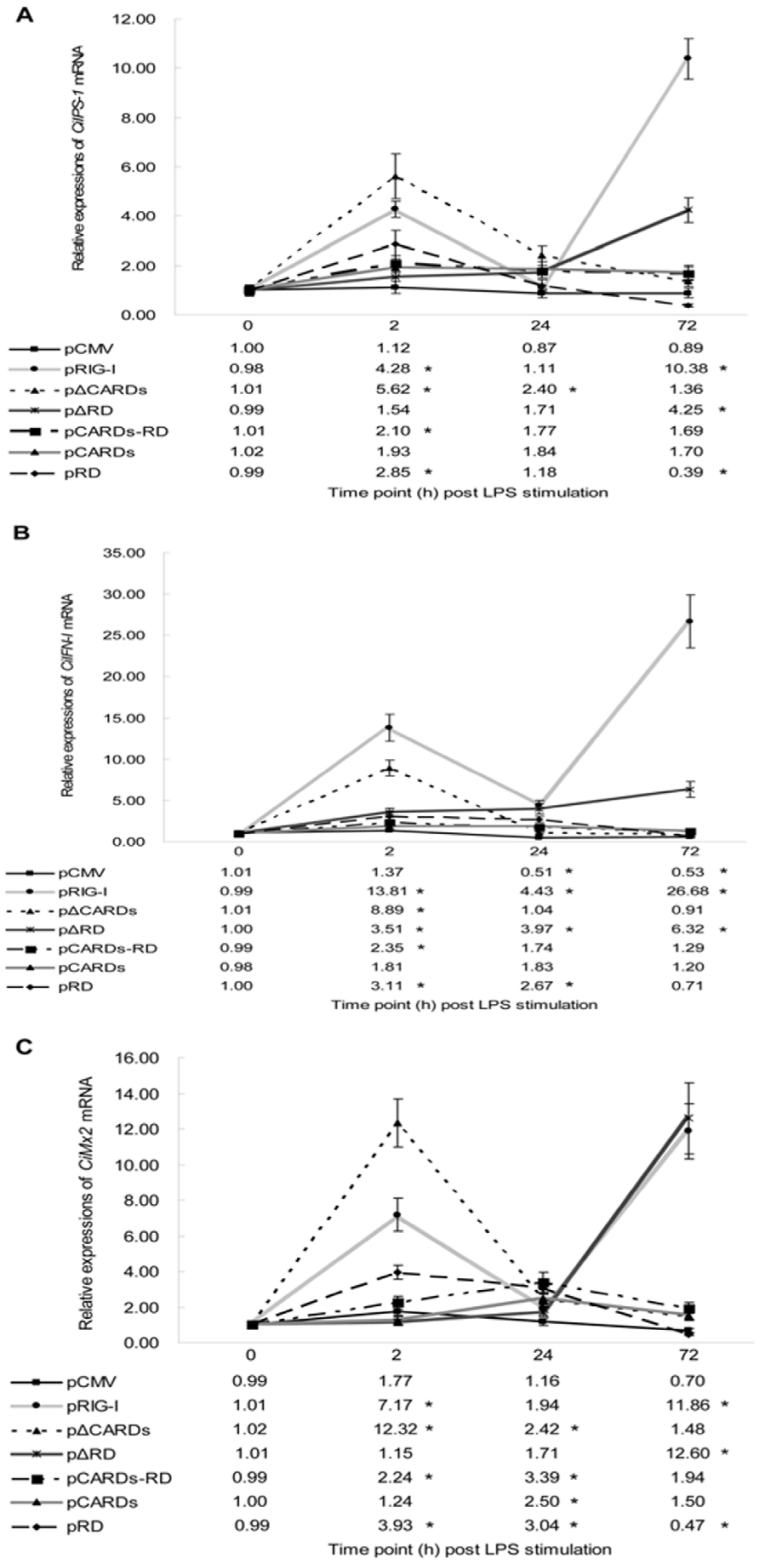
The mRNA expression profiles of several downstream genes of *CiRIG-I* after LPS challenge in seven stable transgenic cells. These genes included *CiIPS-1* (A), *CiIFN-I* (B) and *CiMx2* (C). The concentration of LPS was 10 µg/ml. The mRNA expression was measured at 0, 2, 24 and 72 h. Other captions were referenced as Fig. 4.

To further understanding on the mechanism of innate immune systems of *CiRIG-I* and its domains, the mRNA expression profiles of *CiIPS-1* (Fig. 7A), *CiIFN-I* (Fig. 7B) and *CiMx2* (Fig. 7C) were tested at 0, 2, 24 and 72 h after LPS stimulation. According to the expression patterns of *CiIPS-1* (Fig. 7A), the relative values in cells transfected with pRIG-I were increased at 2 h (4.28 folds) and retrieved the control level at 24 h, then rapidly enhanced at 72 h (10.38 folds). The relative quantities in cells transfected with pΔCARDs began to increase significantly at 2 h (5.62 folds) and declined to 2.40 folds at 24 h, then recovered the control level at 72 h. The expressions in cells transfected with pΔRD were no significant differences at 2 h and 24 h, then enhanced at 72 h (4.25 folds). As for the temporal levels in cells transfected with pCARDs-RD, they were slightly increased at 2 h (2.10 folds), then recovered the control level at 24 h and 72 h. The relative expressions in cells transfected with pCARDs were no significant differences at 2 h, 24 h and 72 h. The relative quantities in cells transfected with pRD were slightly increased at 2 h (2.85 folds) and recovered the control level at 24 h, then decreased at 72 h (0.39 fold).

As for the expression profiles of *CiIFN-I* (Fig. 7B), the relative values in cells transfected with pRIG-I were rapidly increased at 2 h (13.81 folds) and decreased at 24 h (4.43 folds), then were sharply enhanced at 72 h (26.68 folds). The relative quantities in cells transfected with pΔCARDs were significantly increased at 2 h (8.89 folds), and recovered the control level at 24 h and 72 h. The relative expressions in cells transfected with pΔRD were moderately increased, and the relative folds were 3.51 at 2 h, 3.97 at 24 h and 6.32 at 72 h. Similar to the pΔCARDs transfected cells, the relative values in cells transfected with pCARDs-RD were slightly increased at 2 h (2.35 folds), and retrieved the control level at 24 h and 72 h. The expressions in cells transfected with pCARDs were no significant differences at 2 h, 24 h and 72 h. The relative quantities in cells transfected with pRD were slightly enhanced at 2 h (3.11 folds) and 24 h (2.67 folds), then recovered the control level at 72 h.

Next, the temporary expression patterns of *CiMx2* (Fig. 7C) were checked. The relative quantities in cells transfected with pRIG-I were increased at 2 h (7.17 folds) and recovered the control level at 24 h, then sharply enhanced at 72 h (11.86 folds). The relative values in cells transfected with pΔCARDs began to increase significantly at 2 h (12.32 folds), and slightly enhanced at 24 h (2.42 folds), then recovered the control level at 72 h. The relative quantities in cells transfected with pΔRD were no significant differences at 2 h and 24 h, then rapidly enhanced at 72 h (12.60 folds). Furthermore, the relative expressions in cells transfected with pCARDs-RD were slightly increased at 2 h (2.24 folds) and 24 h (3.39 folds), then retrieved the control level at 72 h. The relative values in cells transfected with pCARDs were no significant differences at 2 h, and slightly increased at 24 h (2.50 folds), then retrieved the control level at 72 h. The folds in cells transfected with pRD were 3.93 at 2 h and 3.04 at 24 h, then sharply decreased at 72 h (0.47 fold).

These results demonstrated that helicase domain was pivotal for *RIG-I* signaling cascade and CARDs played an assistant role. In contrast, RD inhibited the activation slightly. Additionally, CARDs and RD alone had little influence upon LPS stimulation.

### Helicase of CiRIG-I Significantly Elicits Signaling Cascade Post PGN Stimulation and CARDs Moderately Strengthen the Function

**Figure 8:**
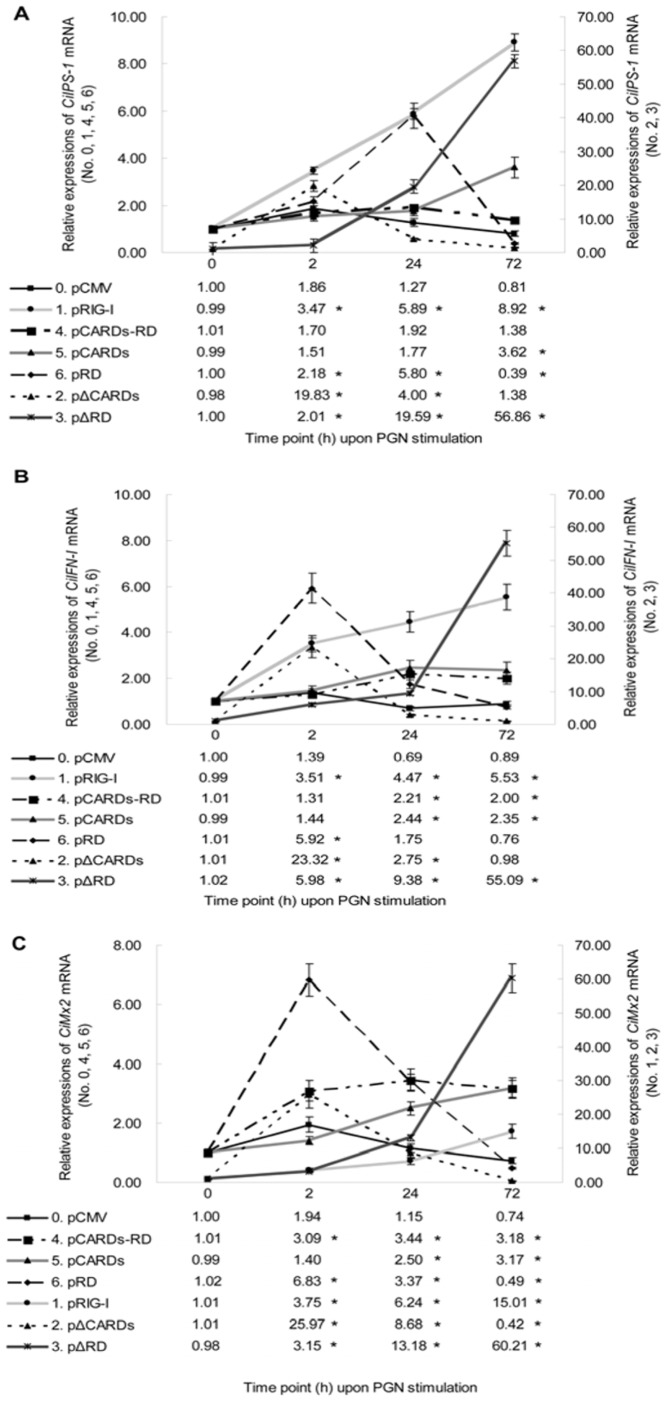
The mRNA expression patterns of several downstream genes of *CiRIG-I* after PGN stimulation in seven stable transgenic cells. These genes included *CiIPS-1* (A), *CiIFN-I* (B) and *CiMx2* (C). The ultimate concentration of PGN was 10 µg/ml. The mRNA expression was measured at 0, 2, 24 and 72 h. Other captions were the same as Fig. 4.

For further clarifying on the mechanisms of *CiRIG-I* and its variants in immune system, the mRNA expressions of *CiIPS-1* (Fig. 8A), *CiIFN-I* (Fig. 8B) and *CiMx2* (Fig. 8C) were tested at 0, 2, 24 and 72 h post PGN stimulation. According to the expression patterns of *CiIPS-1* (Fig. 8A), the relative values in cells transfected with pRIG-I were up-regulated, reached 3.47 folds at 2 h, 5.89 folds at 24 h and 8.92 folds at 72 h compared to the control. The relative quantities in cells transfected with pΔCARDs were significantly increased at 2 h (19.83 folds) and declined to 4.00 folds at 24 h, then recovered the control level at 72 h. The relative expression levels in cells transfected with pΔRD were slightly increased at 2 h (2.01 folds), then sharply enhanced at 24 h (19.59 folds) and 72 h (56.86 folds). The relative values in cells transfected with pCARDs-RD were no significant differences at 2 h, 24 h and 72 h. The relative folds in cells transfected with pCARDs were no significant differences at 2 h and 24 h, then enhanced at 72 h (3.62 folds). The relative quantities in cells transfected with pRD were increased at 2 h (2.18 folds) and 24 h (5.80 folds), then sharply decreased at 72 h (0. 39 fold).

As for the expression profiles of *CiIFN-I* (Fig. 8B), the relative quantities in cells transfected with pRIG-I were up-regulated, reached 3.51 folds at 2 h, 4.47 folds at 24 h and 5.53 folds at 72 h compared to the control. The relative values in cells transfected with pΔCARDs were significantly increased at 2 h (23.32 folds) and rapidly declined at 24 h (2.75 folds), then recovered the control level at 72 h. The relative folds in cells transfected with pΔRD were increased at 2 h (5.98 folds) and 24 h (9.38 folds), and significantly enhanced at 72 h (55.09 folds). The relative quantities in cells transfected with pCARDs-RD were no significant differences at 2 h, and slightly increased at 24 h (2.21 folds) and 72 h (2.00 folds). Similar to the transfected pCARDs-RD cells, the relative values in cells transfected with pCARDs were no significant differences at 2 h, and enhanced at 24 h (2.44 folds) and 72 h (2.35 folds). The relative expressions in cells transfected with pRD were significantly increased at 2 h (5.92 folds), and recovered the control level at 24 h and 72 h.

Next, the temporary expression patterns of *CiMx2* (Fig. 8C) post PGN stimulation were examined. The relative values in cells transfected with pRIG-I reached 3.75 folds at 2 h, 6.24 folds at 24 h and 15.01 folds at 72 h compared to the control. The relative quantities in cells transfected with pΔCARDs began to increase significantly at 2 h (25.97 folds) and declined at 24 h (8.68 folds), then decreased to 0.42 fold at 72 h. The relative folds in cells transfected with pΔRD were increased at 2 h (3.15 folds) and 24 h (13.18 folds), and sharply enhanced at 72 h (60.21 folds). As for the expressions of transfected pCARDs-RD cells, they were up-regulated at 2 h (3.09 folds), and maintained the levels at 24 h (3.44 folds) and 72 h (3.18 folds). The relative values in cells transfected with pCARDs were slightly enhanced at 24 h (2.50 folds) and 72 h (3.17 folds). Finally, the relative quantities in cells transfected with pRD began to increase significantly at 2 h (6.83 folds), and declined to 3.37 folds at 24 h, then recovered at 72 h (0.49 fold).

These results showed helicase played a key function on *RIG-I* signaling cascade and CARDs act as a positive role, whereas RD inhibited the signaling transduction. Additionally, CARDs and RD alone had few effects on PGN stimulation.

## Discussion

*RIG-I* gene is discovered by a group in Shanghai Institute of Hematology in China in 1997. Until recently, *RIG-I* is demonstrated as an essential cytoplasmic component of the sensor for the recognition of intracellular dsRNA analogues (such as poly(I:C)) and various RNA/DNA viruses [13], [35], [36]. In mammals, N-terminal two CARDs are responsible for interacting with downstream signaling pathways that mediate dsRNA inducing *IFN-I* production [37]. The central helicase domain is responsible for dsRNA recognition and binding, which leads to the dimerization and structural alterations of *RIG-I*. Subsequently, *RIG-I* exposes the CARDs to interact with *IPS-1*. The C-terminal RD is tightly auto-regulated, which is mediated by intramolecular interactions with the CARDs. The RD inhibits *RIG-I* signaling in the resting state, however, recent report indicates that RD is responsible for RNA binding during virus infection [22].

Antiviral activity assay showed that apoptosis in overexpression cells of *CiRIG-I* and its domains was delayed more or less. Cells transfected with pΔRD and pRIG-I exhibited a powerful antiviral effect against GCRV (Fig. 3A and Fig. 3B). The relative expressions of downstream genes in cells transfected with pΔRD were up-regulated, and exhibited high levels especially at 48 h including 3.96 folds in *CiIPS-1*, 16.67 folds in *CiIFN-I* and 23.57 folds in *CiMx2*. The cells transfected with pRIG-I and pCARDs also showed a strong mRNA expression of *CiIPS-1*, *CiIFN-I* and *CiMx2* (Fig. 4). In EPC (*Epithelioma papulosum cyprini*) cells, overexpression of the full length of *RIG-I* or CARDs induces the powerful antiviral activity [24]. Similarly, the innate immune response to viral infection in human cells is modified by a functional polymorphism in the CARDs of *RIG-I*, and CARDs alone can activate the signaling cascade leading to *IFN-I* expression in mammal [35], [38]–[40]. Collectively, it implied that CARDs of *RIG-I* were important for antiviral signaling activation in both mammals and teleosts. The cells overexpressing *CiRIG-I* induced a strong antiviral signaling function and transfected pCARDs-RD cells exhibited little regulation post GCRV infection (Fig. 4), which indicated that helicase of *CiRIG-I* played an important role in antiviral immune response in grass carp. Mutation of the ATP-binding site (K270A) inactivates *RIG-I* to trigger antiviral signaling, and the helicase is evidenced to elicit binding of ATPase and *RIG-I* signaling pathway in mammals [15], [35]. In contrast, the functional characterization about helicase of *RIG-I* was not researched in teleosts. Our study provided the evidence that helicase of *CiRIG-I* also played an important role in antiviral signaling channel, which was in consistent with the mammals. Taken together, these results demonstrated CARDs and helicase motifs were crucial for antiviral activity, whereas RD inhibited the activation of signaling cascade in teleosts.

The flag-RIG-I constructs encoding helicase and RD form an inhibitory complex to prevent *RIG-I* signaling pathway in response to Sendai virus in mammals [15], furthermore, RIG-I-ΔCARDs act as a dominant-negative inhibitor of *RIG-I* to bind viral RNA [41]. Unexpectedly, the relative values of *CiIPS-1*, *CiIFN-I* and *CiMx2* mRNA expressions were increased and GCRV quantities were dramatically decreased in transfected pΔCARDs cells (Fig. 4 and Fig. 5). Furthermore, the antiviral activity also showed that ΔCARDs of *CiRIG-I* have ability to delay the cytopathic effect (CPE) (Fig. 3A and Fig. 3B). These data demonstrated that ΔCARDs of *CiRIG-I* were resistant to GCRV replication indeed. It is supposed that there are other mechanisms which elicit an antiviral immune in response to inhibit GCRV replication in teleosts. One possible hypothesis is that the deletion of CARDs may play a role similar to the *LGP2*. Firstly, *LGP2* is induced by GCRV infection in grass carp [42]. Secondly, it is not alone but with a good prototype in Japanese flounder, which demonstrates that mRNA levels of *IFN-I* and *ISGs* (*Mx* and *ISG15*) in the hirame natural embryo cells overexpressing *LGP2* are increased by viral infection [43]. In addition, overexpressing *LGP2* of transfected cells acts as a positive regulator for *IFN-I* production in rainbow trout [44]. The accurate mechanism that ΔCARDs of *CiRIG-I* can elicit an antiviral immune effect to inhibit virus replication needs to be clarified in future.

In accordance with the results of GCRV infection, the cells transfected with pRIG-I and pΔRD showed powerful mRNA expressions of *CiIPS-1*, *CiIFN-I* and *CiMx2* post poly(I:C) stimulation (Fig. 6). The mRNA expressions of *CiIPS-1*, *CiIFN-I* and *CiMx2* increased intermediately in pCARDs transfected cells. The data proved that consensus mechanism for poly(I:C) or GCRV was shown in *RIG-I* signaling pathway. CARDs were crucial for signaling cascade after poly(I:C) stimulation, and the results were uniform in both mammals and teleosts [28], [41]. According to mRNA expressions of *CiIPS-1*, *CiIFN-I* and *CiMx2* in transfected pRD, pΔRD and pRIG-I cells, we concluded that RD played a negative role of the signaling channel. The result was in line with that reported by Hausmann et al. [41]. In mammals, ΔCARDs of *RIG-I* can not induce *IFN-I* effectively and the helicase participates in poly(I:C) inducing *IFN-I* activation [41], [45]. In contrast to the mammals, it was not significant for signaling pathways of *CiRIG-I* helicase, because the relative mRNA expressions of *CiIPS-1*, *CiIFN-I* and *CiMx2* were nearly the same in pCARDs and pΔRD transfected cells. Unexpectedly, the helicase and RD domains (ΔCARDs) triggered a powerful *IPS-1*, *IFN-I* and *Mx2* activation in grass carp. The structure analysis exhibits low homology of helicase and RD domains between grass carp and human [29]. The variant structure of *RIG-I* implies the discrepancy of signaling transduction between mammals and teleosts in the innate immunity [10], [29], [46]. Collectively, these results proved CARDs were crucial for signaling cascade upon poly(I:C) stimulation, and RD played a negative role in the signaling channel. Additionally, helicase and RD had a cooperative effect upon poly(I:C) stimulation, which made up the imperfect theory for the comprehensive mechanism on dsRNA inducing *RIG-I* signaling pathway in vertebrate.

In order to define the regulation of *RIG-I* and its domains to LPS stimulation in grass carp, the temporal expressions of *CiIPS-1*, *CiIFN-I* and *CiMx2* in transfected cells were examined by qRT-PCR (Fig. 7). Based on the common traits, it revealed that helicase motif was pivotal for *RIG-I* signaling transduction and CARDs played an assistant role. In mammals, helicase motif was evidenced for enzymatic activity, ATP hydrolyze, interferon induction, and antiviral signaling [21]. The results indicated that helicase showed a formidable signaling cascade in grass carp coupled with mammals. What puzzled us was that why mRNA levels of *CiIPS-1*, *CiIFN-I* and *CiMx2* sharply decreased at 24 h. To date, LPS is capable of eliciting a wide variety of septic shock, cell injury and lethality [47]. It has long been established that lower vertebrates, most notably fish and amphibians, are resistant to the toxic effect of LPS [48]. So we supposed that LPS just acted as a simple bacterial PAMP at the beginning, as a time-dependent check, the endotoxic effect of LPS was provoked at 24 h, subsequently, some representative domains of *CiRIG-I* could exhibit higher mRNA expression to inhibit the pathogenetic analogue. Although the mechanism of *RIG-I* domain to LPS in signaling transduction was unclear, our results provided a foundation for precise regulation in fish. In combination with mRNA expression profiles in pΔRD and pRD transfected cells, our findings indicated that RD played a suppressive role slightly. Furthermore, RD exhibited a rapid regulation, whereas CARDs played a sustained role in the signaling activation (Fig. 7). In mammals, overexpression of *RIG-I* increases the expression of *IL-1β*, *IL-6* and *IL-8* in gingival fibroblasts after LPS stimulation [49]. Compared with mammals, the lack of *TLR4* ortholog in some fish species leads us to hypothesize that mechanism of LPS recognition in fish may be different from mammals, and zebrafish *TLR4* orthologs negatively regulates the *MyD88*-dependent signaling pathway post LPS stimulation [48]. We also provided the evidence that the signaling pathway of overexpression *RIG-I* was motivated upon LPS stimulation in grass carp. For accurate domain recognition, it was demonstrated that helicase domain was pivotal for signaling cascade and CARDs played an assistant role after LPS stimulation. Interestingly, CARDs and RD alone had little influence post LPS stimulation. Collectively, these results provide the possibility that *CiRIG-I* can function as a pivotal PRR for recognizing gram-negative bacteria pathogens in couple with mammals.

To further clarify functional characterizations of *CiRIG-I* upon PGN stimulation, the mRNA expressions of *CiIPS-1*, *CiIFN-I* and *CiMx2* were detected by qRT-PCR (Fig. 8). According to the common tendency, it proved that helicase was essential for *RIG-I* signaling transduction. Interestingly, the relative expression levels in pRD and pΔCARDs transfected cells reached the maximum at 2 h, then declined sharply. The phenomenon implied that RD excited a rapid regulation upon PGN stimulation. Relatively, the expression patterns of *CiIPS-1*, *CiIFN-I* and *CiMx2* were continuously up-regulated in pRIG-I, pΔRD and pCARDs transfected cells, which indicated that CARDs played positive and persistent roles in *RIG-I* signaling cascade. In the present study, the results clearly suggested that the signaling pathway after *RIG-I* overexpression was activated by PGN stimulation. The data evidenced that gram-positive or gram-negative bacterial analogues could induce *RIG-I* signaling pathway, in which helicase was crucial for signaling cascade. CARDs played a positive role, whereas RD inhibited the activation. Furthermore, RD exhibited a fast regulation and CARDs showed a continuous function. In addition, stronger mRNA levels of *CiIPS-1*, *CiIFN-I* and *CiMx2* were observed post PGN stimulation than those after LPS challenge.

In summary, *RIG-I* exhibits extraordinary broad roles in innate immune responses in teleosts, responding to not only dsRNA virus or synthetic dsRNA but also bacterial PAMPs. The CARDs play a positive function whereas RD shows a repressive role. Helicase domain has pivotal roles in response to dsRNA virus and bacterial PAMPs (LPS and PGN). Interestingly, ΔCARDs display a positive role in *RIG-I* signaling cascade after both virus and PAMPs stimulation.

**Table 1:** Primers used for the construction of vectors and qRT-PCR analyses.

Name	Sequence (5′→3′)	Size (bp)	Application
RF322a	ACTG*GAATTC*ATCGCTGCAAAAatgTACGAG	2896	pRIG-I
		2309	pΔRD
		1390	pCARDs-RD
		797	pCARDs
RR323a	ACTG*GGATCC*TAATGTTGTGTTTGCCGCC	2896	pRIG-I
		2136	pΔCARDs
		1390	pCARDs-RD
		630	pRD
RF477a	ACTG*GAATTC*AAatgTCAGGTGAGATTAAGCTAAGGGA	2136	pΔCARDs
RR478a	ACTG*GGATCC*ctaGGAGATCAGGAAACACCGGC	2309	pΔRD
RF479a	ACTG*GTCGAC*CTGATCTCCAGTAGTAAGGAATGC	1390	pCARDs-RD
RF479b	ACTG*GAATTC*atgCTGATCTCCAGTAGTAAGGAATGC	630	pRD
RR480a	ACTG*GGATCCGTCGAC*CGATCATGCTCCCCTGTG	1390	pCARDs-RD
RR480b	ACTG*GGATCC*tcaCGATCATGCTCCCCTGTG	797	pCARDs
EF125	CGCCAGTGTTGCCTTCGT	99	*EF1α*
ER126	CGCTCAATCTTCCATCCCTT		
IF217	GACCGTAAGAAGTCAGCCTCC	111	*CiIPS-1*
IR218	CCTGAATAACTCTTGATAGCCCTC		
IF590	AAGCAACGAGTCTTTGAGCCT	79	*CiIFN-I*
IR591	GCGTCCTGGAAATGACCT		
MF428	ACATTGACATCGCCACCACT	129	*CiMx2*
MR429	TTCTGACCACCGTCTCCTCC		
VF146	CGAAAACCTACCAGTGGATAATG	135	*VP4*
VR147	CCAGCTAATACGCCAACGAC		

Footnotes: The nucleotides in lowercase mark the initiation codon or the termination codon. The nucleotides with italic type stand for the site of restricted enzyme. “ACTG” in the 5′ terminal represents protective bases.

## Materials and Methods

### Construction of Overexpression Plasmids

The full-length cDNA sequence of *CiRIG-I* was reported previously [29]. The full-length coding sequence of *CiRIG-I* was amplified using LA Taq™ DNA polymerase (TaKaRa, Japan) by primers RF322a and RR323a (Table 1). To generate the corresponding domain vectors (Fig. 1), *CiRIG-I*-ΔCARDs (lack of CARDs), *CiRIG-I*-ΔRD (RD deleted), *CiRIG-I*-CARDs-RD (*CiRIG-I*-CARDs plus *CiRIG-I*-RD with a communal restriction enzyme site, or helicase domain removed), *CiRIG-I*-CARDs (CARDs remained) and *CiRIG-I*-RD (RD preserved) were amplified by PCR. The primer sequences of the variants were listed in Table 1. The corresponding PCR products were ligated into pMD18-T easy vector (TaKaRa), transformed into the competent cells *Escherichia coli* TOP10, and plated on the LB-agar petri-dish containing ampicillin for selection. Colony PCR was used to screen positive colonies. Three of them were picked up and sent to a commercial company (Nanjing Genscript Biotechnology Co., Ltd, China) for sequencing to validate the insert sequences without mutations. The plasmid with correct insert in pMD18-T easy vector was extracted by TIANpure Midi Plasmid Kit (Beijing TIANGEN Biotech, China) and digested with the enzymes of *Eco*RI (Fermentas, Canada) and *Bam*HI (Fermentas), meanwhile, the plasmid of pCMV-EGFP-CMV-SV40 (Fig. 2) was digested with the same enzymes [50]. The target fragments were purified, ligated with T4 DNA ligase (Fermentas), and named as pRIG-I, pΔCARDs, pΔRD, pCARDs-RD, pCARDs and pRD, respectively (the full names were shown in Table 2). As for the construction of pCARDs-RD, the brief procedures as follow: CARDs of *CiRIG-I* were firstly amplified by primers RF322a with *Eco*RI and RR480a with *Sal*I (Fermentas) and *Bam*HI (Table 1) and pCMV-EGFP-CMV-SV40 was digested with *Eco*RI and *Bam*HI, then ligated with T4 DNA ligase. Thus the enzyme cutting site of *Sal*I was introduced. The constructed plasmid above was digested with *Sal*I and *Bam*HI, and RD of *CiRIG-I* was amplified by primers RF479a and RR323a (Table 1), then they were digested with the same enzymes and ligated with T4 DNA ligase. Finally, the plasmid of pCARDs-RD was obtained and validated for sequencing.

**Table 2:** The abbreviation of constructed vectors.

Plasmid name	Abbreviation	Function domains
pCMV-EGFP-CMV-SV40	pCMV	Empty vector
pCMV-EGFP-CMV-SV40-*Ci-RIG-I*	pRIG-I	Full length
pCMV-EGFP-CMV-SV40-*Ci-RIG-I*-ΔCARDs	pΔCARDs	CARDs deleted
pCMV-EGFP-CMV-SV40-*Ci-RIG-I*-ΔRD	pΔRD	RD removed
pCMV-EGFP-CMV-SV40-*Ci-RIG-I*-CARDs-RD	pCARDs-RD	Helicase domain deleted
pCMV-EGFP-CMV-SV40-*Ci-RIG-I*-CARDs	pCARDs	CARDs remained
pCMV-EGFP-CMV-SV40-*Ci-RIG-I*-RD	pRD	RD preserved

### Cells Culture, Plasmids Transfection, Virus Infection and PAMPs Stimulation

CIK cell line, provided by China Center for Type Culture Collection, was grown in DMEM-F12 (Invitrogen, USA) supplemented with 10% fetal bovine serum (FBS; Biosource, USA), 100 IU/ml of penicillin (Sigma, USA) and 100 µg/ml of streptomycin (Sigma) [29]. Cells were incubated at 28°C in a 5% CO_2_ humidified atmosphere. CIK cells were transfected in 6-well plates at a density of 2−5×10^6^ cells/ml with 0.5 µg of purified plasmids by FuGENE® HD Transfection Reagent (Roche, Switzerland) according to the manufacturer’s instruction. After 48 h, cells were cultured in Medium 199 (Sigma) and supplied with 200 µg/ml G-418 (Roche) for 3 weeks of selection. The cells were checked under a fluorescent microscope (Nikon, Japan), and when approximate 50% cells were GFP positive, they were deemed suitable for assessment of the viral infection or PAMPs stimulation. All the following experiments relied on stably transfected CIK cells.

For virus infection, steadily transfected cells including pCMV, pRIG-I, pΔCARDs, pΔRD, pCARDs-RD, pCARDs and pRD were cultured in 24-well plates, they were washed and counted using a hemocytometer, then resuspended into a final concentration of 6×10^5^ cells/ml supplemented with FBS. After 24 h incubation, the cells were washed with phosphate buffer solution (PBS) for three times, and cultured in Medium 199 without FBS. Then they were infected with GCRV (097 strain, 3.63×10^7^ TCID_50_/ml) at a multiplicity of infection (MOI) of 1 [29]. For the time-dependent expression profiles, cells were harvested at 1000 rpm for 8 minutes at 0, 2, 24 and 48 h post infection. RNA was extracted and reverse transcribed.

For PAMPs stimulation, poly(I:C) (Sigma) was dissolved in PBS, then heated to 55°C for 5 minutes and allowed to cool at room temperature. LPS (Sigma) and PGN (Sigma) were also dissolved in PBS following instructions. In accordance with the GCRV infection, the steadily transfected cells were incubated in 24-well plates, and they were treated with 5 µg/ml (terminal concentration) of poly(I:C), 10 µg/ml (terminal concentration) of LPS and PGN, respectively. For studies on mRNA expressions, cells were gathered at 0, 2, 24 and 72 h post stimulation at 1000 rpm for 8 minutes, then RNA was isolated and transcribed.

### Antiviral Activity Assay

The stably transfected cells were seeded into 96-well plates at the density of 4×10^5^ cells/ml, containing 100 µl/well Medium 199 with FBS overnight, then infected with 2-fold-diluted GCRV at the indicated titers in duplicate (Fig. 3B). After 60 h post-infection, cells were fixed with 10% paraformaldehyde for 10 min at room temperature and stained with 0.05% (wt/vol) crystal violet (Sigma) for 30 min. Washed with water and drained, then the plates were photographed under a light box (Bio-Rad, USA). For virus titration test, stably transfected cells were infected with GCRV, and supernatants were harvested at 12 and 48 h post infection, and used for virus titer assay. The virus titer was tested as previous report [51].

### The Temporal Expression Profiles of CiIPS-1, CiIFN-I and CiMx2 after GCRV Challenges

To check the effects of viral infection on *CiRIG-I* signaling pathways in stably transfected cells, qRT-PCR method was established to quantify the mRNA expressions of *CiIPS-1* (accession No., **GQ483645**), *CiIFN-I* (accession No., **DQ357216**) and *CiMx2* (accession No., **AY395698**) post GCRV infection using CFX96 Multicolor Real-time PCR Detection System (Bio-Rad). Three parallel samples from each group were harvested at 0, 2, 24 and 48 h post infection. *EF1α* was utilized as an internal control for cDNA normalization [52]. The primers of *EF1α*, *CiIPS-1*, *CiIFN-I* and *CiMx2* were listed in Table 1 for qRT-PCR. The qRT-PCR and data analysis carried out as previous report [29].

### Comparing the Relative Virus Yields between Transfected pΔCARDs and pCMV Cells Post GCRV Infection

For further research on the antiviral function of pΔCARDs transfected cells, we utilized qRT-PCR to directly quantify the virus yields. The viral expression profiles of *VP4* (segment 6 of GCRV, outer capsid protein, accession No., **GQ469997**) were examined in pΔCARDs and pCMV transfected cells at 2, 24 and 48 h post GCRV infection. The procedures were reported in previous study [29]. The GCRV yields in pΔCARDs tranfected cells were relative to those in pCMV transfected cells (control). The *EF1α* gene was used as an internal control. The data analysis was referenced as above description.

### The mRNA Expression Patterns of CiIPS-1, CiIFN-I and CiMx2 Post Poly(I:C), LPS or PGN Stimulation

To examine the effects of PAMPs (poly(I:C), LPS or PGN) stimulation on *RIG-I* signaling pathway in stably transfected cells, qRT-PCR was employed to quantify the mRNA expressions of *CiIPS-1*, *CiIFN-I* and *CiMx2*. Three independent cell samples from each group were collected at 0, 2, 24 and 72 h post stimulation. The following protocols and data analyses were referenced as above.
